# A comparative analysis of eight machine learning models for the prediction of lateral lymph node metastasis in patients with papillary thyroid carcinoma

**DOI:** 10.3389/fendo.2022.1004913

**Published:** 2022-10-28

**Authors:** Jia-Wei Feng, Jing Ye, Gao-Feng Qi, Li-Zhao Hong, Fei Wang, Sheng-Yong Liu, Yong Jiang

**Affiliations:** The Third Affiliated Hospital of Soochow University, Changzhou First People’s Hospital, Changzhou, Jiangsu, China

**Keywords:** Lateral lymph node metastasis, machine learning, prediction model, random forest, papillary thyroid carcinoma

## Abstract

**Background:**

Lateral lymph node metastasis (LLNM) is a contributor for poor prognosis in papillary thyroid cancer (PTC). We aimed to develop and validate machine learning (ML) algorithms-based models for predicting the risk of LLNM in these patients.

**Methods:**

This is retrospective study comprising 1236 patients who underwent initial thyroid resection at our institution between January 2019 and March 2022. All patients were randomly split into the training dataset (70%) and the validation dataset (30%). Eight ML algorithms, including the Logistic Regression, Gradient Boosting Machine, Extreme Gradient Boosting, Random Forest (RF), Decision Tree, Neural Network, Support Vector Machine and Bayesian Network were used to evaluate the risk of LLNM. The performance of ML models was evaluated by the area under curve (AUC), sensitivity, specificity, and decision curve analysis.

**Results:**

Among the eight ML algorithms, RF had the highest AUC (0.975), with sensitivity and specificity of 0.903 and 0.959, respectively. It was therefore used to develop as prediction model. The diagnostic performance of RF algorithm was dependent on the following nine top-rank variables: central lymph node ratio, size, central lymph node metastasis, number of foci, location, body mass index, aspect ratio, sex and extrathyroidal extension

**Conclusion:**

By combining clinical and sonographic characteristics, ML algorithms can achieve acceptable prediction of LLNM, of which the RF model performs best. ML algorithms can help clinicians to identify the risk probability of LLNM in PTC patients.

## Introduction

Thyroid cancer is one of the most common malignant endocrine carcinomas with a rapidly increasing incidence. Papillary thyroid carcinoma (PTC) is the most common histological type of thyroid cancer (1). The incidence of lymph node metastasis (LNM) is high, ranging from 49% to 90% (2, 3). PTC patients with lateral lymph node metastasis (LLNM) are reported to have higher rates of disease persistence, recurrence and distant metastasis than patients with or without central lymph node metastasis (CLNM) (4).

Current methods for assessing preoperative lymphatic status mainly include ultrasound and fine needle aspiration cytology (FNAC). However, the diagnostic sensitivity of ultrasound to cervical LNM is only about 20% to 40% (5, 6). And the false negative rate of FNAC can be as high as 16.7% (7). Hence, occult LLNM has been reported to occur in up to 55% of PTC patients with clinically negative (cN0) lateral neck (8). Prophylactic lateral neck dissection (LND) is not recommended for patients with cN0 lateral neck (9–11). Considering the existence of occult LLNM that is not easily detected preoperatively, some patients undergoing thyroidectomy may have some metastatic lymph nodes in the lateral compartment (12). Therefore, accurate assessment of lateral cervical lymph node status in PTC patients has a guiding role in clinical decision-making.

At present, studies have reported several risk factors of LLNM, and established predictive models. However, these results are inconsistent. Due to the complexity of medical data, there are significant connections between the various factors of predictive models, Therefore, there are also significant differences in the calculation methods of the model. Machine learning (ML) is a new type of artificial intelligence and is widely used in healthcare data analysis (13–17). By using the ML algorithms, data can be accurately processed, connections among important data can be analyzed, and accurate decisions can be made. Through the powerful predictive capabilities of ML algorithms, predictive tools that are better than traditional statistical modeling can be developed in some cases. Unfortunately, there are currently no studies training ML algorithms to predict LLNM of PTC patients.

We aimed to develop models based on eight ML algorithms using clinical and sonographical features. By selecting one model that performs best in predicting the risk of LLNM among PTC patients, individual strategies could be proposed to help clinicians to make therapeutic decisions.

## Materials and methods

### Patients population

This retrospective study was approved by the Ethics Committee of Changzhou First People’s Hospital, and written informed consent was obtained from all patients. Consecutive patients who underwent initial thyroid resection at our institution between January 2019 and March 2022 were reviewed. The exclusion criteria were as follows: (1) non-PTCs or other subtypes than classic PTC; (2) history of prior treatment for head and neck cancer; (3) history of cervical radiation exposure in childhood; (4) family history of thyroid cancer; (5) history with other malignancy; (6) incomplete clinical data; (7) loss to follow-up; (8) patients who underwent non-curative surgery (residual tumor or lymph node detected within 6 months of initial surgery). A total of 1236 patients were enrolled in this study.

### Surgical strategy

All patients were confirmed as Bethesda Categories V or VI according to ultrasound-guided FNAC. Cervical lymph nodes with the following characteristics were suspected of metastases: hyperechoic changes, roundness or necrosis, loss of the fatty hilum, microcalcification or peripheral vascularity (18). FNAC was performed preoperatively to confirm the histopathological diagnosis of suspicious lateral lymph nodes.

All patients underwent total thyroidectomy or thyroid lobectomy. According to the Chinese guidelines for diagnosis and treatment of differentiated thyroid carcinoma, central neck dissection (CND) was routinely performed for PTC patients. According to the American Thyroid Association guidelines (9) and Chinese guidelines, LND was performed only in patients with high suspicion of LLNM based on preoperative imaging data and FNAC. CND referred to the removal of prelaryngeal, pretracheal and paratracheal lymph nodes. LND included the removal of the lateral lymph nodes, including level II to V, while preserving the spinal accessory nerve, internal jugular vein, or sternocleidomastoid muscle.

### Clinicopathological and sonographical features

We included a total of 18 variables in this study. Clinicopathological features included sex, age, body mass index (BMI), diabetes, BRAF V600E mutation, chronic lymphocytic thyroiditis (CLT), maximum tumor size, the number of foci, bilaterality, location, CLNM and central lymph node ratio (CLNR). BMI (kg/m^2^) was defined as weight (kg) divided by height (m) squared. According to the World Health Organization-BMI standard, enrolled PTC patients were divided into normal (BMI < 25 kg/m^2^), overweight (25 kg/m^2^ ≤ BMI). The diagnosis of CLT included any of the following: (i) antibodies to thyroid peroxidase level >50 IU/mL, (ii) diffuse heterogeneity on ultrasound, (iii) diffuse lymphocytic thyroiditis on histopathology (19). CLNR was defined as the ratio of metastatic lymph nodes in the central compartment out of the number of dissected lymph nodes in the central compartment.

Specific evaluation parameters of malignant thyroid lesions included: nodular composition, echogenicity, calcification, aspect ratio and margin, including irregular shape and extrathyroidal extension (ETE). More than two radiologists with 10 years of experience in thyroid cancer ultrasound diagnosis evaluated images.

The surgeon dissected all lymph node specimens according to the level of the neck and sent them to the department of pathology for examination. Each lymph node was fixed in 20% buffered formalin, embedded in paraffin, sectioned, and stained with hematoxylin and eosin. Lymph nodes with suspected cancer involvement were further investigated by using immunohistochemical staining. All pathological specimens were reviewed and cross-checked by two or more experienced pathologists microscopically.

### Development of ML-based models

We split all patients randomly into two groups, the training dataset (70%) and the validation dataset (30%). Based on the presence or absence of LLNM, We also divided the overall study population into two groups and compared baseline information. Logistic Regression (LR) was conducted to assess independent predictors associating with LLNM.

Eight types of ML algorithms were applied in this study, including LR, Gradient Boosting Machine (GBM), Extreme Gradient Boosting (XGB), Random Forest (RF), Decision Tree (DT), Neural Network (NNET), Support Vector Machine (SVM) and Bayesian Network (BN) (16, 17, 20–22). Only LR is considered as conventional method among all eight algorithms, and the others are representative supervised ML-based algorithms. Only DT and LR are explainable, where the users are able to identify the function between variables and predicted outcomes. The other algorithms are inexplicable, where function between variables and the outcome is invisible to the user. In order to construct more reliable ML-based predictive models, we used the z- score normalization to preprocess all continuous variables (23).

### Validation strategy and feature selection

Overfitting, meaning the model becomes too specific to fit to another dataset, is a common risk, especially when the number of variables is large (24). In order to minimize the adverse effect of overfitting, we adopted 5-fold cross-validation in the training set. The relative importance ranking of each input variable was analyzed in each model. We compared all variables to determine their predictive importance for LLNM. The predictive performance of these models was assessed by the area under the receiver operating characteristic (ROC) curve (AUC). In the comparison of ML algorithms, the closer the AUC was to 1, the better the performance of the model. However, ROC curve is a traditional diagnostic method that focuses only on sensitivity and specificity. In this case, we employed decision curve analysis (DCA) to assess the clinical utility of these models (25).

### Statistical analysis

All statistical analysis was performed by using SPSS Version 25.0 software (Chicago, IL, USA), and R software Version 3.5.3 (The R Foundation for Statistical Computing). Pearson Chi-square test or Fisher’s exact test was used for categorical data. Normally distributed quantitative parameters were compared by Student’s t-test, while non- normally distributed parameters were compared by the Mann-Whitney U test. We considered *P* value <0.05 to be statistically significant. For independent risk factors for LLNM, odds ratios (ORs) with 95% confidence intervals (CIs) were calculated by using multivariate logistic regression analysis with backward stepwise selection. R software (Version 3.5.3) was used to develop ML-based models and DCA.

## Results

### Demographics and sonographic features

The 1236 patients were divided into two groups randomly: approximately 866 (70%) cases were conducted as the training dataset, and the remaining around 370 (30%) cases were used as the validation dataset. No significant differences were observed in clinicopathological and sonographic features of thyroid nodules (*P >*0.05 for all comparisons), which justified their use as training and validation cohorts (**Table 1**).

**Table 1:** Comparison of clinical and ultrasonic characteristics of the PTC patients in the training and validation dataset.

Characteristics	Total	Training dataset	Validation dataset	*P* value
	n=1236	n=866	n=370	
Sex				
Male	350 (28.3%)	257 (29.7%)	93 (25.1%)	
Female	886 (71.7%)	609 (70.3%)	277 (74.9%)	0.105
Age (Y)				
≥55	226 (18.3%)	157 (18.1%)	69 (18.6%)	
<55	1010 (81.7%)	709 (81.9%)	301 (81.4%)	0.829
BMI (kg/m^2^)				
Normal	797 (64.5%)	552 (63.7%)	245 (66.2%)	
Overweight	439 (35.5%)	314 (36.3%)	125 (33.8%)	0.405
Diabetes				
Absence	1050 (85.0%)	739 (85.3%)	311 (84.1%)	
Presence	186 (15.0%)	127 (14.7%)	59 (15.9%)	0.564
BRAF V600E mutation				
Negative	141 (11.4%)	93 (10.7%)	48 (13.0%)	
Positive	1095 (88.6%)	773 (89.3%)	322 (87.0%)	0.258
CLT				
Presence	397 (32.1%)	269 (31.1%)	128 (34.6%)	
Absence	839 (67.9%)	597 (68.9%)	242 (65.4%)	0.223
Maximum tumor size (cm)				
≤1	726 (58.7%)	507 (58.5%)	219 (59.2%)	
>1 to ≤2	345 (27.9%)	239 (27.6%)	106 (28.6%)	
>2 to ≤4	137 (11.1%)	101 (11.7%)	36 (9.7%)	
>4	28 (2.3%)	19 (2.2%)	9 (2.4%)	0.787
The number of foci				
1	831 (67.2%)	582 (67.2%)	249 (67.3%)	
2	272 (22.0%)	189 (21.8%)	83 (22.4%)	
3 or more	133 (10.8%)	95 (11.0%)	38 (10.3%)	0.922
Bilaterality				
Absence	981 (79.4%)	684 (79.0%)	297 (80.3%)	
Presence	255 (20.6%)	182 (21.0%)	73 (19.7%)	0.609
Location				
Middle/Lower	637 (51.5%)	434 (50.1%)	203 (54.9%)	
Upper	599 (48.5%)	432 (49.9%)	167 (45.1%)	0.126
Nodular composition				
Mixed cystic and solid	10 (0.8%)	7 (0.8%)	3 (0.8%)	
Solid	1226 (99.2%)	859 (99.2%)	367 (99.2%)	0.996
Hypoechogenicity				
Absence	53 (4.3%)	41 (4.7%)	12 (3.2%)	
Presence	1183 (95.7%)	825 (95.3%)	358 (96.8%)	0.236
A/T				
≤1	441 (35.7%)	311 (35.9%)	130 (35.1%)	
>1	795 (64.3%)	555 (64.1%)	240 (64.9%)	0.794
Irregular shape				
Absence	926 (74.9%)	653 (75.4%)	273 (73.8%)	
Presence	310 (25.1%)	213 (24.6%)	97 (26.2%)	0.547
ETE				
Absence	1097 (88.8%)	770 (88.9%)	327 (88.4%)	
Presence	139 (11.2%)	96 (11.1%)	43 (11.6%)	0.785
Microcalcification				
Absence	440 (35.6%)	309 (35.7%)	131 (35.4%)	
Presence	796 (64.4%)	557 (64.3%)	239 (64.6%)	0.926
CLNM				
Absence	565 (45.7%)	385 (44.5%)	180 (48.6%)	
Presence	671 (54.3%)	481 (55.5%)	190 (51.4%)	0.176
CLNR				
<0.5	914 (73.9%)	630 (72.7%)	284 (76.8%)	
≥0.5	322 (26.1%)	236 (27.3%)	86 (23.2%)	0.141
LLNM				
Absence	996 (80.6%)	690 (79.7%)	306 (82.7%)	
Presence	240 (19.4%)	176 (20.3%)	64 (17.3%)	0.218

PTC, papillary thyroid carcinoma; Y, year; BMI, body mass index; CLT, chronic lymphocytic thyroiditis; A/T, aspect ratio (height divided by width on transverse views); ETE, extrathyroidal extension; CLNM, central lymph node metastasis; CLNR, central lymph node ratio; LLNM, lateral lymph node metastasis.

Among the 866 patients in the training cohort, 257 were males and 609 were females. The average age was 45.1 ± 10.5 years (range 18–82 years). Four hundred and eighty-one (55.5%) patients developed CLNM, and 176 (20.3%) patients developed LLNM. The validation cohort consisted of 370 patients (mean age, 46.3 ± 11.2 years), including 93 males and 277 females. CLNM were positive in 190 (51.4%) cases, and LLNM were positive in 64 (17.3%) cases. Baseline epidemiological and sonographic characteristics of the two cohorts are shown in **Table 1**.

### Univariate and multivariate analysis of potential factors for LLNM

In univariable analysis, gender, diabetes, tumor size, number of foci, bilaterality, location, aspect ratio, irregular shape, ETE, microcalcification, CLNM and CLNR were all significantly related with LLNM in all patients (all *P* < 0.05).

All above parameters were included in the LR. The results showed that male (OR: 1.521, 95% CI: 1.077–2.149, *P*=0.017), tumor size ranges between 1.0 to 2.0 cm (OR: 1.753, 95% CI: 1.206–2.548, *P*=0.003), tumor size ranges between 2.0 to 4.0 cm (OR: 3.381, 95% CI: 2.075–5.507, *P <*0.001), tumor size > 4.0 cm (OR: 2.167, 95% CI: 1.015–5.625, *P*=0.012), three or more tumor foci (OR: 3.254, 95% CI: 2.014–5.257, *P <*0.001), tumors located in the upper pole (OR: 2.368, 95% CI: 1.691–3.317, *P*<0.001), presence of ETE (OR: 9.145, 95% CI: 4.092–20.439, *P*<0.001), presence of CLNM (OR: 4.261, 95% CI: 2.637–6.887, *P*<0.001), and CLNR ≥0.5 (OR: 2.379, 95% CI: 1.642–3.449, *P*<0.001) were independent predictors of LLNM (**Table 2**).

**Table 2:** Univariate analysis and multivariate analysis of factors associated with LLNM in whole cohort.

Characteristics	LLNM, No. (%)			Multivariate analysis	
	Presence (n=240)	Absence(n=996)	*P* value	Adjusted OR (95% CI)	*P* value
Sex					
Female	141 (58.8%)	745 (74.8%)		Ref	
Male	99 (41.3%)	251 (25.2%)	<0.001	1.521 (1.077–2.149)	0.017
Age (Y)					
≥55	36 (15.0%)	190 (19.1%)			
<55	204 (85.0%)	806 (80.9%)	0.142		
BMI (kg/m^2^)					
Normal	143 (59.6%)	654 (65.7%)			
Overweight	97 (40.4%)	342 (34.3%)	0.077		
Diabetes					
Presence	26 (10.8%)	160 (16.1%)		Ref	
Absence	214 (89.2%)	836 (83.9%)	0.042	1.395 (0.833–2.336)	0.206
BRAF V600E mutation					
Negative	29 (12.1%)	112 (11.2%)			
Positive	211 (87.9%)	884 (88.8%)	0.714		
CLT					
Presence	78 (32.5%)	319 (32.0%)			
Absence	162 (67.5%)	677 (68.0%)	0.888		
Maximum tumor size (cm)					
≤1	80 (33.3%)	646 (64.9%)		Ref	
>1 to ≤2	99 (41.3%)	246 (24.7%)		1.753 (1.206–2.548)	0.003
>2 to ≤4	52 (21.7%)	85 (8.5%)		3.381 (2.075–5.507)	<0.001
>4	9 (3.8%)	19 (1.9%)	<0.001	2.167 (1.015–5.625)	0.012
The number of foci					
1	137 (57.1%)	694 (69.7%)		Ref	
2	49 (20.4%)	223 (22.4%)		1.073 (0.712–1.616)	0.737
3 or more	54 (22.5%)	79 (7.9%)	<0.001	3.254 (2.014–5.257)	<0.001
Bilaterality					
Absence	174 (72.5%)	807 (81.0%)		Ref	
Presence	66 (27.5%)	189 (19.0%)	0.003	1.474 (0.832–2.610)	0.184
Location					
Middle/Lower	79 (32.9%)	558 (56.0%)		Ref	
Upper	161 (67.1%)	438 (44.0%)	<0.001	2.368 (1.691–3.317)	<0.001
Nodular composition					
Mixed cystic and solid	2 (0.8%)	8 (0.8%)			
Solid	238 (99.2%)	988 (99.2%)	0.963		
Hypoechogenicity					
Absence	11 (4.6%)	42 (4.2%)			
Presence	229 (95.4%)	954 (95.8%)	0.801		
A/T					
≤1	66 (27.5%)	375 (37.7%)		Ref	
>1	174 (72.5%)	621 (62.3%)	0.003	1.091 (0.773–1.539)	0.621
Irregular shape					
Absence	160 (66.7%)	766 (76.9%)		Ref	
Presence	80 (33.3%)	230 (23.1%)	0.001	1.016 (0.701–1.473)	0.933
ETE					
Absence	192 (80.0%)	905 (90.9%)		Ref	
Presence	48 (20.0%)	91 (9.1%)	<0.001	9.145 (4.092–20.439)	<0.001
Microcalcification					
Absence	63 (26.3%)	377 (37.9%)		Ref	
Presence	177 (73.8%)	619 (62.1%)	<0.001	1.305 (0.904–1.883)	0.156
CLNM					
Absence	28 (11.7%)	537 (53.9%)		Ref	
Presence	212 (88.3%)	459 (46.1%)	<0.001	4.261 (2.637–6.887)	<0.001
CLNR					
<0.5	104 (43.3%)	810 (81.3%)		Ref	
≥0.5	136 (56.7%)	186 (18.7%)	<0.001	2.379 (1.642–3.449)	<0.001

PTC, papillary thyroid carcinoma; Y, year; BMI, body mass index; CLT, chronic lymphocytic thyroiditis; A/T, aspect ratio (height divided by width on transverse views); ETE, extrathyroidal extension; CLNM, central lymph node metastasis; CLNR, central lymph node ratio; LLNM, lateral lymph node metastasis.

### Predictive performance and clinical usefulness of ML-based models

**Figure 1:**
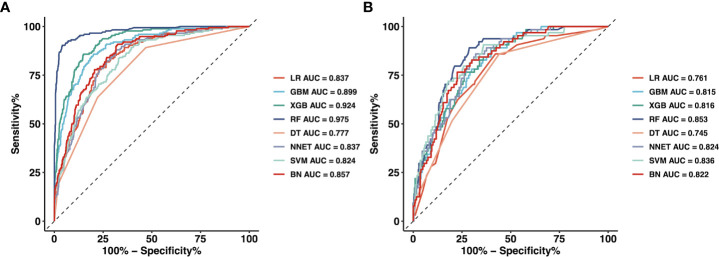
The mixed ROC curves of the eight machine learning models for prediction of LLNM. **(A)** The mixed ROC curves in the training cohort; **(B)** The mixed ROC curves in the validation cohort. *ROC* receiver operating characteristic; *LLNM* Lateral lymph node metastasis; *LR* Logistic Regression; *GBM* Gradient Boosting Machine; *XGB* Extreme Gradient Boosting; *RF* Random Forest; *DT* Decision Tree; *NNET* Neural Network, *SVM* Support Vector Machine; *BN* Bayesian Network.

We used 18 variables to develop predictive models for LLNM based on eight algorithms. **Figure 1** and **Table 3** show the predictive performance of these models. In the training cohort, the best performance was observed in the RF model, whose AUC was 0.975 (**Figure 1A**). It was followed by XGB and GBM with AUCs of 0.924 and 0.899, respectively. All ML-based models except DT (AUC=0.777) and SVM (AUC=0.824) were better than the conventional method–LR (AUC=0.837). In the training cohort, the RF model performed the best with an AUC as high as 0.853 (**Figure 1B**). And the sensitivity and specificity of the RF model in the training cohort were 0.903 and 0.959, respectively. The sensitivity and specificity of the RF model in the validation cohort were 0.891 and 0.775, respectively (**Table 3**). Above results proved the best diagnostic performance of RF model.

**Table 3:** Predictive performance comparison of the eight types of machine learning algorithms in the training and validation dataset.

Methods	AUC	Sensitivity	Specificity
Training dataset			
LR	0.837	0.818	0.733
GBM	0.899	0.858	0.800
XGB	0.924	0.858	0.851
RF	0.975	0.903	0.959
DT	0.777	0.892	0.530
NNET	0.837	0.881	0.683
SVM	0.824	0.835	0.671
BN	0.857	0.901	0.681
Validation dataset			
LR	0.761	0.859	0.578
GBM	0.815	0.891	0.601
XGB	0.816	0.766	0.732
RF	0.853	0.891	0.775
DT	0.745	0.859	0.559
NNET	0.824	0.859	0.680
SVM	0.836	0.906	0.641
BN	0.822	0.762	0.712

AUC, the area under the curve; LR, logistic regression; GBM, gradient boosting machine; XGB, extreme gradient boosting; RF, random forest; DT, decision tree; NNET, neural network; SVM, support vector machine; BN, Bayesian network.

**Figure 2:**
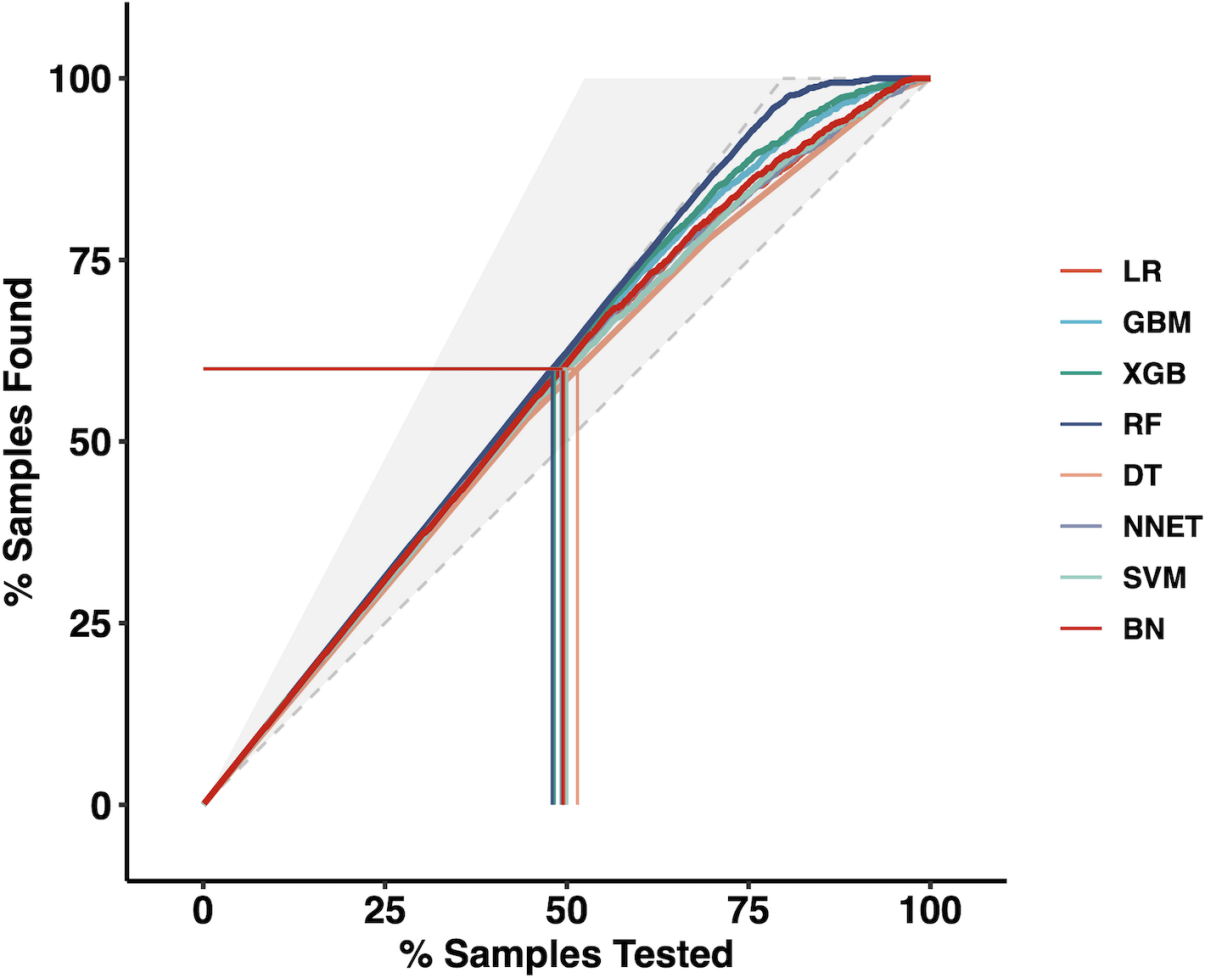
The mixed Lift curves of the eight machine learning models in the training cohort. *LR* Logistic Regression; *GBM* Gradient Boosting Machine; *XGB* Extreme Gradient Boosting; *RF* Random Forest; *DT* Decision Tree; *NNET* Neural Network, *SVM* Support Vector Machine; *BN* Bayesian Network.

Moreover, we applied the mixed Lift curves of the eight ML models in the training cohort. The drawing process of the Lift curve is similar to the ROC curve, the difference is that the Lift value and the robust plane pose change in opposite directions, forming the opposite form of the Lift curve and the ROC curve. Furthermore, the Lift curve considers the accuracy of the classifier: the ratio of the number of positive classes obtained with the classifier to the number of positive classes obtained randomly without the classifier. RF model also has the best diagnostic performance among the current mix Lift curves (**Figure 2**).

**Figure 3:**
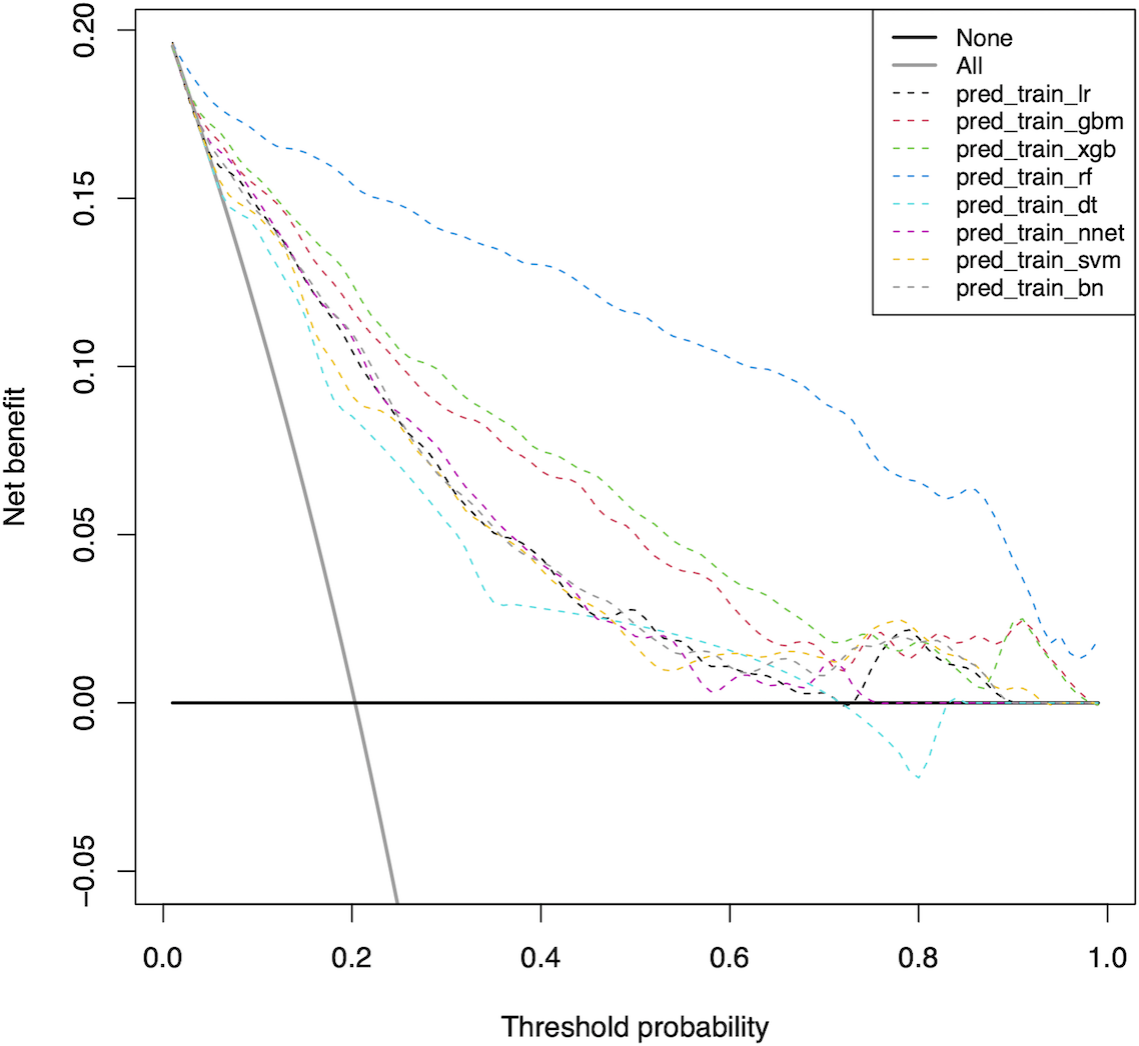
Decision curve for predictive models based on machine learning models in the training cohort. *LR* Logistic Regression; *GBM* Gradient Boosting Machine; *XGB* Extreme Gradient Boosting; *RF* Random Forest; *DT* Decision Tree; *NNET* Neural Network, *SVM* Support Vector Machine; *BN* Bayesian Network.

Furthermore, we used the DCA to evaluate the clinical values of these models (**Figure 3**). Assuming that all patients do not have positive lymph nodes in the latter compartment, the solid black line (negative line) indicates that when no patient accepts LND, net benefit is zero. On the contrary, the solid grey line (positive line) indicates the net benefits when all patients have LLNM and receive LND. According to the incidence of LLNM among patients with PTC, the reasonable range of thresholds was set from 0.3 to 0.9. Almost at the entire range, all ML-based models showed higher net benefits than the two extreme lines (negative line and positive line) except DT. It was noteworthy that RF, XGB and GBM performed significantly better than the others at most of threshold points. Within a threshold range of 0 to 0.7, XGB had a higher net benefit than GBM. But within the threshold range of 0.8 to 0.9, the net benefit of GBM was higher than that of XGB. In almost the entire threshold probability range, the RF model had the highest net benefit, much higher than XGB and GBM.

### Relative importance of variables in ML-based models

**Figure 4:**
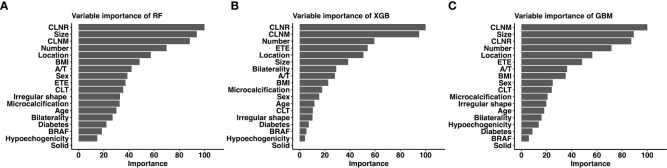
Relative importance ranking of each input variable for prediction of LLNM in the machine learning models. **(A)** Random Forest; **(B)** Gradient Boosting Machine; **(C)** Extreme Gradient Boosting.

Considering favorable AUCs and clinical benefits based on the DCA, we selected RF, XGB and GBM as the models with the most potential for predicting LLNM in PTC patients. The relative importance of variables in RF, XGB and GBM for predicting LLNM is shown in **Figure 4**. Although the importance of variables in these ML algorithms were slightly different among these three models. It was obvious that CLNR, CLNM, size, number of foci and location ranked in the top five. In contrast, solid, hypoechogenicity, BRFA, and diabetes did not contribute much to the prediction of the risk of LLNM in PTC patients.

**Figure 5:**
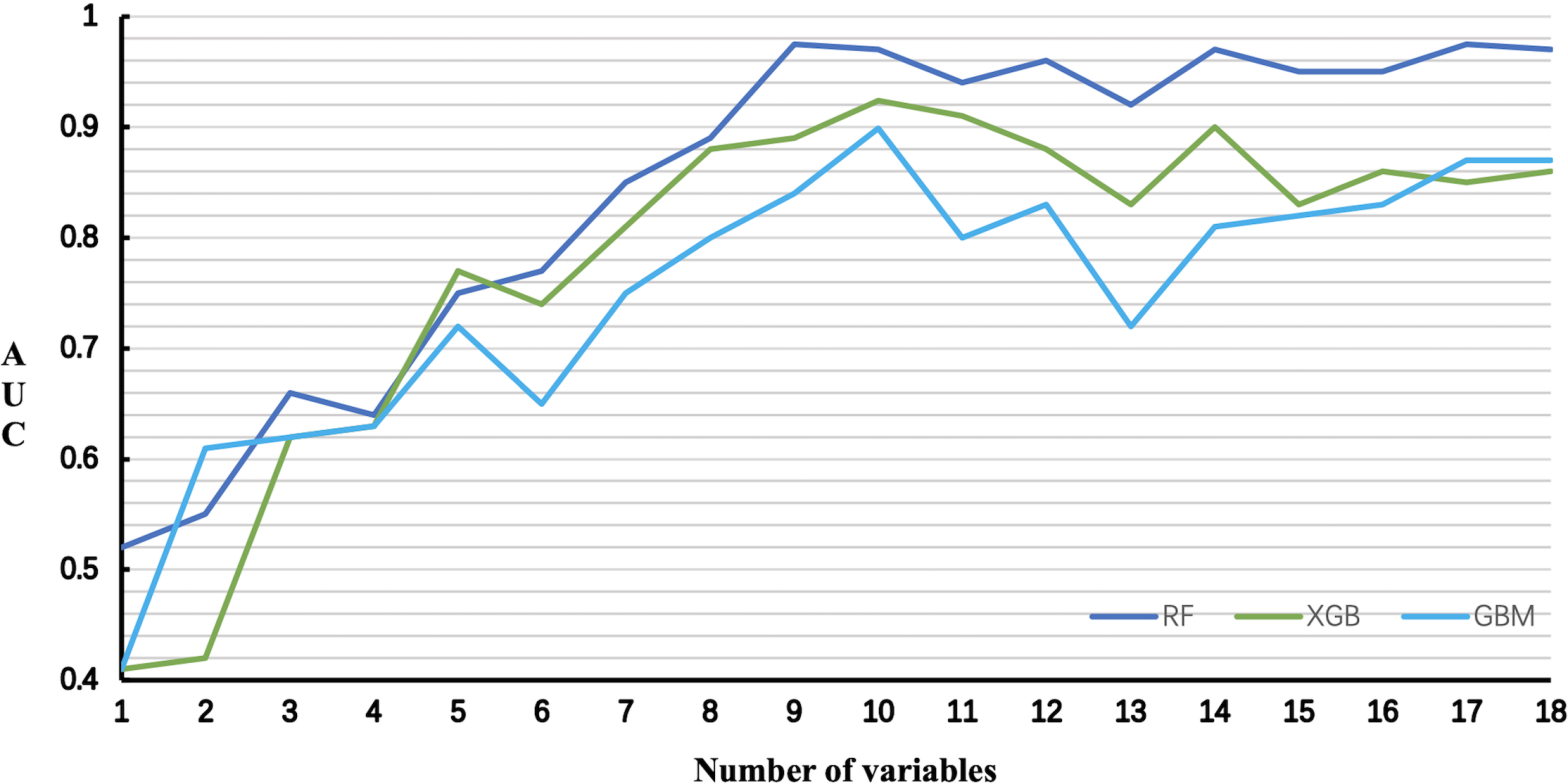
Predictive performance of the RF, XGB and GBM model with different numbers of variables. *RF* Random Forest; *XGB* Extreme Gradient Boosting; *GBM* Gradient Boosting Machine.

The relationship between the number of variables and the AUCs of models is shown in **Figure 5**. The AUC of the RF model plateaued when 9 variables were introduced, while the AUCs of XGB and GBM started to decrease when they reached the highest point (10 variables).

Accordingly, we chose the RF model as the best predictive model according to its best performance in ROC curve, Lift curve and DCA. We further performed the collinearity test for nine top-rank variables in RF model. In general, the variance inflation factor (VIF) and tolerance are most commonly used to detect collinearity. Tolerance and VIF are reciprocal of each other. When the VIF is less than 3, there is no collinearity problem; when the VIF value is greater than 3 and less than 10, there is a moderate degree of collinearity; when the VIF is greater than 10, there is a serious collinearity problem. Likewise, tolerance values greater than 0.1 indicate no collinearity. In our study, the VIF for CLNR, size, CLNM, number of foci, location, BMI, aspect ratio, sex and ETE were 1.493, 1.160, 1.513, 1.042, 1.031, 1.055, 1.049, 1.113 and 1.021, respectively. Moreover, the tolerance value for CLNR, size, CLNM, number of foci, location, BMI, aspect ratio, sex and ETE were 0.670, 0.862, 0.661, 0.960, 0.970, 0.948, 0.953, 0.898 and 0.979, respectively. The above results indicate that there is no collinearity between the above variables.

At last, The nine top-rank variables were identified to construct the best predictive model, including CLNR, size, CLNM, number of foci, location, BMI, aspect ratio, sex and ETE.

## Discussion

Although lateral neck is the second most common compartment for LNM, prophylactic LND was not recommended by most clinical guidelines, including National Comprehensive Cancer Network (11), American Thyroid Association guidelines (9), the Japanese Association of Endocrine Surgeons and the Japanese Society of Thyroid Surgeons (10). And the indication for prophylactic LND remained controversial (26). Moreover, due to the low metastasis rate in this region as well as the high incidence of postoperative complications, LND is performed only for those with clinically positive LLNM in most medical institutions.

ML algorithms have the advantage of automatically learning from input data and identifying patterns and trends in these data. There are many studies using ML for the differential diagnosis of benign and malignant thyroid nodules (27, 28). However, there are few studies on the application of ML models to predict LNM in PTC patients, especially LLNM. Lee et al. (29) applied a deep learning-based computer-aided diagnosis system to locate and diagnose metastatic lymph nodes in patients with thyroid cancer. But they used only one ML model and did not compare the performance of multiple ML models in distinguishing metastatic lymph nodes in patients with thyroid cancer.

We aimed to predict the risk of LLNM more accurately and filter the best prediction model. In this study, by combining the clinical and imaging characteristics of patients, we developed eight models using the ML algorithm to predict the LLNM of PTC patients. We first used ROC analysis and mixed Lift curves to evaluate the predictive performance of these models. Most of the eight models maintained high AUCs. Except for DT and SVM, all ML-based models performed better than LR model using a traditional statistical method in predicting LLNM (**Figures 1**, **2** and **Table 3**). The clinical value of these models was then assessed using DCA. DCA has enormous clinical utility and has been used in many medical studies. According to DCA, most of these models outperformed the positive line and negative line, indicating that the overall net benefit of performing LND for patients with high risk of LLNM identified by the model was higher than in all patients or no patient undergoing the same surgical procedure. Three models (RF, XGB and GBM) performed better than the others at most of threshold points. At last, combined with the results of ROC, mixed Lift curves and DCA, the RF model performed best in distinguishing between LLNM and non-LLNM. Besides, the validation set confirmed that the RF model was the best predictive model for LLNM of PTC (AUC=0.853).

The RF structure is simple, easy to understand, and more efficient than similar methods. From a computational point of view, RF is a more advanced algorithm based on DT, which can be used for both regression and classification. In addition, RF can be directly used for high-dimensional problems. From a statistical point of view, RF has the following characteristics, that is, the priority of characteristics, different weight coefficients fall into different categories, and illustration and unsupervised learning ability. Moreover, RF is a well-known ML algorithm for classification tasks and is inherently capable of resisting overfitting. According to previous meta-analysis of metastatic lymph node studies (30), computed tomography demonstrated a sensitivity of 81.1% and a specificity of 84.0% in detecting LLNM, and ultrasound demonstrated a sensitivity of 75.8% and a specificity of 88.0%. When we compared the diagnostic performance of the RF model with that in the meta-analysis, our RF model achieved better sensitivity (90.3%) and specificity (95.9%).

Although the link between variables and outcomes in most ML-based models is invisible, the predicted importance of variables in each model could be obtained by using a classifier-specific estimator (**Figure 4**). Therefore, the nine top-rank variables were considered to be the most important risk factors for LLNM in the RF model: CLNR, size, CLNM, number of foci, location, BMI, aspect ratio, sex and ETE. We also tested the collinearity of these variables and found that there was no collinearity between the above variables. Tumor size is the most important preoperative predictor of LLNM in PTC patients, and CLNR is the most important postoperative predictor of LLNM in PTC patients. Diameter was also reported in previous studies to be an independent risk factor for LLNM in PTC patients (31, 32). This may be attributed to the more extensive the tumors, the more aggressive and proliferative. Lymph node ratio is considered a variable reflecting tumor burden in PTC and other solid tumors (33). It is important to note that LNR is not only affected by disease burden, but also by the extent of neck dissection and pathological examination. This requires the surgeon to remove the central lymph nodes as much as possible and the pathologist to carefully check the status of the removed lymph nodes ensuring the accuracy of the model. According to RF model, for patients with several preoperative risk factors of LLNM, detailed preoperative examinations (such as high-resolution ultrasound by experienced sonographers) should be performed to detect small metastatic lymph nodes early. Moreover, experienced surgeons are recommended to perform detailed operations for these patients, and carbon nanoparticles suspension injection can be used during the operation to prevent the miss of small metastatic lymph nodes. Furthermore, for patients with high CLNR, heightened vigilance for occult LLNM may be warranted for these patients postoperatively. More closely follow-up should be applied for these patients after surgery. For suspicious lymph nodes detected in the lateral compartment postoperatively, FNAC should be actively conducted to confirm the histopathologic diagnosis, and LND should be considered if necessary.

To our surprise, BMI had no significant significance in univariate analysis (*P*=0.077), but was ranked sixth in the top. In addition, although the seventh-ranked shape (A/T) was statistically significant in univariate variables, it had no significant significance in multivariate analysis. This may be attributed to the amazing advantages of ML-based models in data mining, which can find more relationships between variables and results than traditional methods. Because LR is used to analyze prognostic factors based on linear combinations between variables, if the degree of correlation between variables is high, the analysis is limited by overfitting results. ML models, on the other hand, do not assume a linear combination of variables used, thus reducing the effect of correlations between variables. Therefore, factors including BMI and aspect ratio were important constituent variables of RF models and were used in other ML models at high frequencies. Thus, the AUC of the LR model based on the above factors was significantly lower than the most ML-based models.

The advantage of this research lies in the innovation of technology and method. By using eight ML methods, we outperformed other methods on clinical data and its application. However, there also has limitations. First, because this was a retrospective study, potential selection bias might exist. Second, ML model we built was based on the data from a single institution, which may limit its generality. Moreover, patients enrolled in our study were all native Chinese population, most of whom were female. Residual confounding variables of unmeasured factors such as race and region cannot be ruled out. Last, the AUCs of the training set were higher than that of the validation set, indicating the overfitting in ML algorithms-based models. This may be related to the fact that our models are complicated and the description data is too accurate. We will conduct prospective multicenter institutional trials to achieve more objective conclusions by increasing the amount of data, reducing the number of data characteristics (dimension), and reducing the complexity of the models to reduce overfitting.

In summary, we proved that ML algorithms are feasible to incorporate clinicopathological and sonographic features to predict LLNM in patients with PTC. We used the ML algorithms to construct and compare the performance of eight predictive models, of which the RF model is the best. In the future, we will integrate imaging, molecular, and genetic data to improve the performance of our models, thus providing more accurate methods for clinical and surgical decisions and postoperative follow-up.

## Data availability statement

The raw data supporting the conclusions of this article will be made available by the authors, without undue reservation.

## Ethics statement

Written informed consent was obtained from the individual(s) for the publication of any potentially identifiable images or data included in this article.

## Author contributions

J-WF and L-ZH: writing - original draft, software, and data curation. S-YL: validation, formal analysis, and data curation. FW: conceptualization. JY and G-FQ: validation and investigation. YJ: writing - review & editing, visualization, and supervision. All authors contributed to the article and approved the submitted version.

## Acknowledgments

Lei Qin, the English language editor, was responsible for correcting language and grammar issues.

## Conflict of interest

The authors declare that the research was conducted in the absence of any commercial or financial relationships that could be construed as a potential conflict of interest.

## Publisher’s note

All claims expressed in this article are solely those of the authors and do not necessarily represent those of their affiliated organizations, or those of the publisher, the editors and the reviewers. Any product that may be evaluated in this article, or claim that may be made by its manufacturer, is not guaranteed or endorsed by the publisher.

## References

[1] Scheffel RS Dora JM Maia AL. BRAF mutations in thyroid cancer. Curr Opin Oncol (2022) 34(1):9–18. doi: 10.1097/CCO.0000000000000797 34636352

[2] Huang Y Yin Y Zhou W. Risk factors for central and lateral lymph node metastases in patients with papillary thyroid micro-carcinoma: Retrospective analysis on 484 cases. Front Endocrinol (Lausanne) (2021) 12:640565. doi: 10.3389/fendo.2021.640565 33746905 PMC7973362

[3] Feng JW Qu Z Qin AC Pan H Ye J Jiang Y. Significance of multifocality in papillary thyroid carcinoma. Eur J Surg Oncol (2020) 46(10 Pt A):1820–8. doi: 10.1016/j.ejso.2020.06.015 32732090

[4] Feng JW Yang XH Wu BQ Sun DL Jiang Y Qu Z. Predictive factors for central lymph node and lateral cervical lymph node metastases in papillary thyroid carcinoma. Clin Transl Oncol (2019) 21(11):1482–91. doi: 10.1007/s12094-019-02076-0 30879178

[5] Alabousi M Alabousi A Adham S Pozdnyakov A Ramadan S Chaudhari H. Diagnostic test accuracy of ultrasonography vs computed tomography for papillary thyroid cancer cervical lymph node metastasis: A systematic review and meta-analysis. JAMA Otolaryngol Head Neck Surg (2022) 148(2):107–18. doi: 10.1001/jamaoto.2021.3387 PMC8613701 34817554

[6] Xu SY Yao JJ Zhou W Chen L Zhan WW. Clinical characteristics and ultrasonographic features for predicting central lymph node metastasis in clinically node-negative papillary thyroid carcinoma without capsule invasion. Head Neck (2019) 41(11):3984–91. doi: 10.1002/hed.25941 31463972

[7] Paek SH Kim BS Kang KH Kim HS. False-negative BRAF V600E mutation results on fine-needle aspiration cytology of papillary thyroid carcinoma. World J Surg Oncol (2017) 15(1):202. doi: 10.1186/s12957-017-1266-5 29132392 PMC5683441

[8] Lim YS Lee JC Lee YS Lee BJ Wang SG Son SM. Lateral cervical lymph node metastases from papillary thyroid carcinoma: predictive factors of nodal metastasis. Surgery (2011) 150(1):116–21. doi: 10.1016/j.surg.2011.02.003 21507446

[9] Haugen BR Alexander EK Bible KC Doherty GM Mandel SJ Nikiforov YE. American Thyroid association management guidelines for adult patients with thyroid nodules and differentiated thyroid cancer: The American thyroid association guidelines task force on thyroid nodules and differentiated thyroid cancer. Thyroid (2015) 26(1):1–133. doi: 10.1089/thy.2015.0020 PMC4739132 26462967

[10] Takami H Ito Y Okamoto T Yoshida A. Therapeutic strategy for differentiated thyroid carcinoma in Japan based on a newly established guideline managed by Japanese society of thyroid surgeons and Japanese association of endocrine surgeons. World J Surg (2011) 35(1):111–21. doi: 10.1007/s00268-010-0832-6 21042913

[11] Haddad RI Nasr C Bischoff L Busaidy NL Byrd D Callender G. NCCN guidelines insights: Thyroid carcinoma, version 2.2018. J Natl Compr Canc Netw (2018) 16(12):1429–40. doi: 10.6004/jnccn.2018.0089 30545990

[12] Zhan S Luo D Ge W Zhang B Wang T. Clinicopathological predictors of occult lateral neck lymph node metastasis in papillary thyroid cancer: A meta-analysis. Head Neck (2019) 41(7):2441–9. doi: 10.1002/hed.25762 30938923

[13] West E Mutasa S Zhu Z Ha R. Global trend in artificial intelligence-based publications in radiology from 2000 to 2018. AJR Am J Roentgenol (2019) 213(6):1204–6. doi: 10.2214/AJR.19.21346 31414886

[14] Wu Y Rao K Liu J Han C Gong L Chong Y. Machine learning algorithms for the prediction of central lymph node metastasis in patients with papillary thyroid cancer. Front Endocrinol (Lausanne) (2020) 11:577537. doi: 10.3389/fendo.2020.577537 33193092 PMC7609926

[15] Enrico C. Precision oncology: The promise of big data and the legacy of small data. Front ICT (2017) 4:22. doi: 10.3389/fict.2017.00022

[16] Dominietto MD Capobianco E. Expected impacts of connected multimodal imaging in precision oncology. Front Pharmacol (2016) 7:451. doi: 10.3389/fphar.2016.00451 27965577 PMC5126138

[17] Capobianco E. Systems and precision medicine approaches to diabetes heterogeneity: a big data perspective. Clin Transl Med (2017) 6(1):23. doi: 10.1186/s40169-017-0155-4 28744848 PMC5526830

[18] Wang Y Chen M Chen P Tong J Zhang Y Yang G. Diagnostic performance of ultrasound and computed tomography in parallel for the diagnosis of lymph node metastasis in patients with thyroid cancer: a systematic review and meta-analysis. Gland Surg (2022) 11(7):1212–23. doi: 10.21037/gs-22-347 PMC9346219 35935558

[19] Grani G Carbotta G Nesca A D'Alessandri M Vitale M Del Sordo M. A comprehensive score to diagnose hashimoto's thyroiditis: a proposal. Endocrine (2015) 49(2):361–5. doi: 10.1007/s12020-014-0441-5 25280964

[20] Gonzalez GH Tahsin T Goodale BC Greene AC Greene CS. Recent advances and emerging applications in text and data mining for biomedical discovery. Brief Bioinform (2016) 17(1):33–42. doi: 10.1093/bib/bbv087 26420781 PMC4719073

[21] Ngiam KY Khor IW. Big data and machine learning algorithms for health-care delivery. Lancet Oncol (2019) 20(5):e262–73. doi: 10.1016/S1470-2045(19)30149-4 31044724

[22] Zhu J Zheng J Li L Huang R Ren H Wang D. Application of machine learning algorithms to predict central lymph node metastasis in T1-T2, non-invasive, and clinically node negative papillary thyroid carcinoma. Front Med (Lausanne) (2021) 8:635771. doi: 10.3389/fmed.2021.635771 33768105 PMC7986413

[23] Shalabi LA Shaaban Z Kasasbeh B. Data mining: A preprocessing engine. J Comput Sci (2006) 2(9):735–9. doi: 10.3844/jcssp.2006.735.739

[24] Jung Y. Multiple predicting K-fold cross-validation for model selection. J nonparametr Stat (2018) 30(1/2):197–215. doi: 10.1080/10485252.2017.1404598

[25] Van Calster B Wynants L Verbeek JFM Verbakel JY Christodoulou E Vickers AJ. Reporting and interpreting decision curve analysis: A guide for investigators. Eur Urol (2018) 74(6):796–804. doi: 10.1016/j.eururo.2018.08.038 30241973 PMC6261531

[26] White C Weinstein MC Fingeret AL Randolph GW Miyauchi A Ito Y. Is less more? a microsimulation model comparing cost-effectiveness of the revised American thyroid association's 2015 to 2009 guidelines for the management of patients with thyroid nodules and differentiated thyroid cancer. Ann Surg (2020) 271(4):765–73. doi: 10.1097/SLA.0000000000003074 30339630

[27] Daniels K Gummadi S Zhu Z Wang S Patel J Swendseid B. Machine learning by ultrasonography for genetic risk stratification of thyroid nodules. JAMA Otolaryngol Head Neck Surg (2020) 146(1):36–41. doi: 10.1001/jamaoto.2019.3073 31647509 PMC6813575

[28] Zhao CK Ren TT Yin YF Shi H Wang HX Zhou BY. A comparative analysis of two machine learning-based diagnostic patterns with thyroid imaging reporting and data system for thyroid nodules: Diagnostic performance and unnecessary biopsy rate. Thyroid (2021) 31(3):470–81. doi: 10.1089/thy.2020.0305 32781915

[29] Lee JH Baek JH Kim JH Shim WH Chung SR Choi YJ. Deep learning-based computer-aided diagnosis system for localization and diagnosis of metastatic lymph nodes on ultrasound: A pilot study. Thyroid (2018) 28(10):1332–8. doi: 10.1089/thy.2018.0082 30132411

[30] Xing Z Qiu Y Yang Q Yu Y Liu J Fei Y. Thyroid cancer neck lymph nodes metastasis: Meta-analysis of US and CT diagnosis. Eur J Radiol (2020) 129:109103. doi: 10.1016/j.ejrad.2020.109103 32574937

[31] Zhang X Chen W Fang Q Fan J Feng L Guo L. Lateral lymph node metastases in T1a papillary thyroid carcinoma: Stratification by tumor location and size. Front Endocrinol (Lausanne) (2021) 12:716082. doi: 10.3389/fendo.2021.716082 34335480 PMC8320373

[32] Song J Yan T Qiu W Fan Y Yang Z. Clinical analysis of risk factors for cervical lymph node metastasis in papillary thyroid microcarcinoma: A retrospective study of 3686 patients. Cancer Manag Res (2020) 12:2523–30. doi: 10.2147/CMAR.S250163 PMC7153998 32308489

[33] Yu ST Ge JN Sun BH Wei ZG Xiao ZZ Zhang ZC. Lymph node yield in the initial central neck dissection (CND) associated with the risk of recurrence in papillary thyroid cancer: A reoperative CND cohort study. Oral Oncol (2021) 123:105567. doi: 10.1016/j.oraloncology.2021.105567 34710736

